# Explaining the experience of breastfeeding in women with gestational diabetes and designing and implementing an educational program based on planned behavior theory: a combined exploratory study protocol

**DOI:** 10.1186/s12978-024-01751-y

**Published:** 2024-02-05

**Authors:** Soltan Asghari, Sanaz Mollazadeh, Nahid Jahani shoorab, Smira Shahbazzadegan, Talat Khadivzadeh

**Affiliations:** 1 Department of Midwifery, Research Student Committee, Mashhad University of Medical Sciences, Mashhad, Iran; 2 Nursing and Midwifery Care Research Center, Mashhad University of Medical Sciences, Mashhad, Iran; 3 Department of Midwifery, School of Nursing and Midwifery, Ardabil University of Medical Sciences, Ardabil, Iran

**Keywords:** Breastfeeding, Gestational diabetes, The theory of planned behavior

## Abstract

**Background:**

Gestational diabetes is a type of carbohydrate intolerance that is diagnosed for the first time during pregnancy. Researches have shown that gestational diabetes is associated with many negative prenatal and birth outcomes. Because of the complications such as infant death, several diabetics’ mothers plan to stop breastfeeding. Research findings indicate a decrease in breastfeeding in mothers with gestational diabetes/ or contradictory tissues regarding the factors affecting the breastfeeding behavior of mothers with gestational diabetes and a special program to promote breastfeeding for these mothers based on the social and cultural conditions of Iranian society. The present study aims to design an interventional program with a mixed qualitative study based on the theory of planned behavior (PBT) to prevent the decrease of breastfeeding in diabetic mothers.

**Methods/design:**

A mixed methods exploratory design will be used to conduct this study in 3 phases. The first phase (qualitative): The purpose of the first phase is to understand the experience of breastfeeding mothers who had gestational diabetes, which will be done using the directed content analysis method. The purposive sampling will be used on pregnant mothers with gestational diabetes 30–34 weeks and mothers with infants (up to 6 months) with a history of gestational diabetes. The second phase include designing and implementing an educational program based on the PBT: Education will be conducted based on the needs assessment of the qualitative phase, the opinions of the focus group, and the literature review, then the breastfeeding behavior will be measured using the survey tool of “breastfeeding drop”. The third phase: Interventional quantitative phase: The sample size will be carried out by a pilot study, then a designed program as an educational intervention for teaching breastfeeding behavior based on the PBT for 30–34 weeks pregnant mothers with gestational diabetes will be implemented during 3–4 sessions and breastfeeding behavior will be evaluated after delivery.

**Discussion:**

This is the first mixed-method study in Iran that led to implement an interventional program based on the theory of planned behavior. Because of the complications such as infant death, several diabetics’ mothers plan to stop breastfeeding. We hope that the result of this research will be a step in solving breastfeeding problems in mothers with gestational diabetes.

## Plain language summary

Gestational diabetes is a type of carbohydrate intolerance diagnosed for the first time during pregnancy. The rate of gestational diabetes has increased along with the increase in the prevalence of diabetes risk factors. It is associated with multiple prenatal and birth outcomes. Despite the incomparable benefits of breastfeeding providing health to gestational diabetes mothers, the rate of breastfeeding is low. This is the first study in Iran using a mixed method approach in 3 phases to implement an educational interventional program with a qualitative study directed by theory of planned behavior (TPB) to prevent the decrease of breastfeeding in mothers with gestational diabetes. The purpose of first phase of study (qualitative) is to understand women’s experience of gestational diabetes, which will be done using the directed content analysis method. The purposive sampling will be used for pregnant mothers with gestational diabetes at 30–34 weeks and mothers with infants (up to 6 months). The second phase is designing and implementing an educational program based on the PBT: Educational aims and content will be prepared based on the need achieved in the qualitative phase, the opinions of the focus group with experts? Mothers? Whom? And the literature review, then the breastfeeding behavior will be measured using the survey tool “breastfeeding drop”. The third phase includes: Interventional quantitative phase an educational program will be implemented for training breastfeeding behavior based on the theory of PBT for 30–34 weeks pregnant mothers with gestational diabetes. This program will be administered as an educational intervention during 3–4 sessions and breastfeeding behavior will be evaluated after delivery.

## Background

Gestational diabetes is a type of carbohydrate intolerance that occurs with different severity, diagnosed for the first time during pregnancy. The rate of gestational diabetes has increased along with the increase in the prevalence of diabetes risk factors such as obesity, increasing maternal age, family history of diabetes [1]. The International Diabetes Federation in 2017 estimated that 21.3 million of all live births were belong to women who had some form of hyperglycemia during pregnancy. It is estimated that 86.4% of these cases were due to gestational diabetes [2]. Research has shown that gestational diabetes is associated with multiple negative prenatal and birth outcomes, including preeclampsia, fetal macrosomia, and shoulder dystocia, followed by an increase in the rate of instrumental and cesarean delivery, and birth injuries. In addition, gestational diabetes is associated with an increased risk of long-term adverse outcomes, including type 2 diabetes in the mother in the future, increased obesity, diabetes, and cardiovascular disease in the infant [3]. One of the complications of gestational diabetes is breastfeeding failure. Despite the incomparable benefits of breast milk in providing the health of mother and infant, in mothers with gestational diabetes, the rate of breastfeeding is low. Admission to NICU, delay in lactogenesis II or incomplete information affect mothers’ breastfeeding behavior. There is no specific prescripted plan for breastfeeding mothers [4, 5].

This shows the necessity of investigating the experiences of mothers with gestational diabetes from breastfeeding in different sociocultural contexts. The result of Negvin. et al. study (2019) showed that the risk of early cessation of breastfeeding 12 months after delivery is higher in mothers with gestational diabetes, Also they conducted a one-year prospective study on 2030 Vietnamese mothers with gestational diabetes to investigate the relationship between gestational diabetes and the length of breastfeeding after giving birth. They reported that the relative risk of stopping breastfeeding in mothers with gestational diabetes was much higher compared to healthy mothers who were examined in the first, third, sixth, and 12th months after delivery [5]. The result of Herbert et al. study (2021) showed that gestational diabetes was more common in American Indian, Alaska Natives, and Hispanic women than in black and non-Hispanic women (12% vs. 9%), and those with gestational diabetes had the lowest rates of exclusive breastfeeding (51% vs. 57–69%) compared to other women without gestational diabetes [4]. The results of many studies showed that stopping breastfeeding early was associated with many complications for the mother and the baby [6–18]. The results of Chertok et al. study (2016) showed that there is a relationship between breastfeeding self-efficacy and the breastfeeding behavior of mothers. They showed that breastfeeding self-efficacy can be increased by encouraging and supporting breastfeeding by health care providers and facilitating early and continuous breastfeeding. Early breastfeeding support is important among women who are at increased risk for delayed initiation of lactation or delayed lactogenesis II, such as women with gestational diabetes [19]. The results of qualitative studies showed that women with diabetes emphasized the need for continuous counseling about breastfeeding and education as well as help to solve breastfeeding challenges [20, 21]. Considering the incomparable benefits of breastfeeding for both mother and baby, management and follow-up of breastfeeding for mothers with gestational diabetes should be one of the basic challenges for every society. Women with gestational diabetes need ongoing support after hospital discharge to maintain long-term breastfeeding [5].

According to the theory of planned behavior, a person who has a high understanding of control over his behavior and has the intention to perform that behavior will most likely do it. Perceived control depends on the presence or absence of facilitators or obstacles to perform a behavior and the perceived power of the individual. In the presence of a high amount of control beliefs about the existence of a facilitator for a behavior, the perceived power of the person and the possibility of the occurrence of the behavior increases. Based on this, the purpose of this research is to explain the understanding and experiences of breastfeeding in women with gestational diabetes and to determine the effect of implementing an educational intervention based on the theory of planned behavior. This research aims to solve breastfeeding problems in mothers with gestational diabetes.

### Study aim

By achieving the results of this study, it is possible to get a deep insights and detailed information about the understanding of diabetic mothers about maintaining breastfeeding according to their needs and expectations, as a result, to explain the breastfeeding experience of mothers with gestational diabetes and to design and implement an educational program and evaluate the its’ effects on mothers’ breastfeeding behavior after delivery.

### Specific objectives

The general purpose of this mixed study is to explain the breastfeeding experiences of women with gestational diabetes and to design and implement an educational program using the theory of planned behavior and evaluate the effects of it on mothers’ breastfeeding behavior after delivery.

The overall goal of the first qualitative phase: is to gain understand and experiences of breastfeeding women with gestational diabetes.

### Specific objectives of the qualitative stage

1. Explanation of breastfeeding behavior of women with gestational diabetes
2. Explaining the perceived behavioral beliefs of women with gestational diabetes about breastfeeding behavior
3. Explaining the perceived subjective norms of women with gestational diabetes about breastfeeding behavior
4. Explanation of perceived control beliefs of women with gestational diabetes from breastfeeding behavior
5. Explaining the behavioral intention of women with gestational diabetes regarding breastfeeding

The general goal of the second quantitative stage: designing an educational program based on the theory of planned behavior.

Specific Objectives of the second phase of educational program design:

1. Assessment and prioritization of the educational needs of women with gestational diabetes
2. Determining the goals and activities of the educational program for women with gestational diabetes.
3. Evaluation design of educational program designed for women with gestational diabetes

The overall goal of the third quantitative phase: determine the effect of the implementation of the educational program based on the theory of planned behavior on the breastfeeding behavior of women with gestational diabetes.

The specific objectives of the third phase are to determine the effectiveness of the implementation of the educational program: which will be measured based on the “breastfeeding drop prediction tool”.

1. Determining the comparison of breastfeeding behavior of women with gestational diabetes before and after the implementation of the intervention
2. Determining the comparison of breastfeeding intention of women with gestational diabetes before and after the implementation of the intervention

## Methods/design

A mixed methods exploratory design will be used to conduct this study in 3 phases. The first phase (qualitative): The purpose of the first phase is to understand women’s experience of gestational diabetes, which will be done using the directed content analysis method. Breastfeeding in mothers with gestational diabetes is a multidimensional concept that is rooted in the subject of mothers and forms in the context of a society with the cultural complexities of that society. Breastfeeding behavior is the result of a mother’s interaction with herself and her world. In addition to attitudes, this behavior is influenced by important others and the control of a mother’s perceived behavior, therefore, for a deep understanding of this phenomenon and to explain the concept of understanding and breastfeeding experiences of women with gestational diabetes, a directed qualitative approach based on the theory of planned behavior will be used.

The second phase: Designing and implementing an educational program based on the theory of planned behavior: From the findings of the qualitative phase, the educational needs of participating mothers with gestational diabetes will be extracted. At this phase, observation, in-depth interviews, and interviews with key and knowledgeable participants will be used as techniques for assessing the needs.

### Sample size and sampling method

Following the approval of the research project by the Ethics Committee of Mashhad University of Medical Sciences, the purposive sampling (pregnant mothers with gestational diabetes 30–34 weeks and mothers with infants (up to 6 months) with a history of gestational diabetes in the last pregnancy) with maximum diversity in terms of mother’s age, mother’s education, infant’s age, and breastfeeding experience, after stating the objectives of the research and obtaining informed consent, women will enter the research and the necessary coordination will be made regarding the appropriate time and place for the interview. Women who are willing and able to explain their understanding and experience of menopause symptoms and its effect on life will be selected and the interviews of this stage will begin. In the in-depth interview approach, the interview begins with an open question and is followed by targeted questions related to the predicted classes of the theory of planned behavior. The qualitative phase of the current study includes the needs assessment of mothers with a history of gestational diabetes, which is the first step in designing an educational program, along with a literature review. At this stage, the researcher went to hospitals, comprehensive health centers, and health care centers and conducted semi-structured, in-depth interviews with mothers who have the conditions to describe their experiences and perceptions. To gain their trust, they will ask to express their needs, opinions, and beliefs in the field of breastfeeding in simple language. In this way, their experiences, behavioral beliefs, subjective beliefs, and control beliefs in the field of breastfeeding behavior will explain to complete the matrix of various structures of the theory of planned behavior (attitude, subjective norms, perceived behavioral control, intention, and behavior). In qualitative research, it is not possible to determine the sample size in advance and it is determined during the study. Interviews will continue until reaching data saturation or when no new data is available to complete and enrich the classes developed during the research.

### Inclusion criteria

In the qualitative part of the study, the mothers with infants under one year of age with a history of gestational diabetes in the last pregnancy under the coverage of comprehensive health centers who do not have any other medical diseases and are willing to participate in the study will participate by obtaining written consent.

### Data analysis

Qualitative content analysis, which is used to mentally interpret the content of written data, identifies codes and themes through a systematic classification process [22]. In this research, there is a pre-determined format and matrix based on the structures of the theory of planned behavior (attitude, subjective norms, perceived behavioral control, intention, and behavior), and content analysis with an inductive approach based on the structures of this theory will be done. Therefore, directed content analysis will be used to perform the analysis. Data analysis in mixed-method research is considered to be a part of a cyclical four-step process (information collection, data analysis, data interpretation, and validation) whose steps affect each other [23]. Obviously, in this study, data collected through interviews, group discussions, and continuous comparison methods is used for data analysis. With the arrival of new data, the process of data comparison and classification will continue with new codes extracted and integrated into the previous data. With the progress of research, interviews, and observations become more focused. The circular approach and continuous backward and forward along with constant comparison and data collection and classification will explain the main concepts, theoretical relations, and processes and finally identify the main variable and the base on the theory. Therefore, in the current research, data collection and interpretation will be done in parallel. We will use four criteria to evaluate the accuracy of the qualitative data (Credibility, Dependability, Confirmability, Transferability [24]. The interview texts and codes will organize in MAXQDA.

#### Data connection and integration

In this mixed study, the researcher will use data connection and integration. It means that the findings of the qualitative parts of the study will be used in the direction of revision (compilation of the educational program for mothers with gestational diabetes) based on the theory of planned behavior. Therefore, in addition to the fact that the data of each of the qualitative and quantitative stages will be analyzed separately, by summarizing the results of the qualitative and quantitative stages, the educational needs of mothers will be identified and prioritized, and an educational program based on the theory of planned behavior will be designed and implemented [25].

#### The second step, designing the educational program

The educational program based on the constructs of the theory of planned behavior will include designing program goals, implementation, and evaluation. The design of educational intervention can be divided into two basic steps:

A. Determining goals (by experienced experts)

After the needs of mothers with gestational diabetes were extracted and listed through qualitative data, and literature review, the needs of mothers with gestational diabetes will be prioritized as follows. First, to prioritize the needs of mothers with gestational diabetes, criteria will be considered that include the prevalence of the problem, the severity of the problem, the ability to intervene (can the problem be solved with the available resources?), the government’s concern (do planners and policymakers want to solve the problem?) and society (do the individuals involved in this issue want to deal with the problem?). The mentioned criteria will provide the possibility of selecting priority problems in a ranked table (Likert 1 to 5). For prioritization, the opinions of 6 to 8 experts composed of neonatologists, gynecologists, and health education specialists in various fields of reproductive health, breastfeeding, and midwifery education and one diabetic mother and experienced experts will be participated, then needs that can be planned and implemented will be prioritized and selected. In this regard, a focused group will be discussed with interested and experienced experts in various health fields. The focus group approach is a very efficient and useful method for extracting different ideas and opinions and pondering them. This approach is a type of group discussion, the main purpose of which is to identify different points of view on the topic under discussion and to understand the issues from the perspective of different individuals [26]. In this study, the research team, using the results of a qualitative study and a literature review and searching in reliable scientific databases and line with the existing instructions and guidelines, will pay the general and specific goals of the educational program based on the priority needs of mothers with gestational diabetes.

B. Elaboration of educational intervention

The results obtained in the previous phases will reveal which structures (attitude, subjective norms, perceived behavioral control, intention, and behavior) have the highest priority as the focus of intervention planning. In addition to the results of the qualitative part of the study, the results of the pre-test will show which structures have the highest and lowest scores, which structures have the most problems, and which structures are more important for learning. Based on the results obtained from the qualitative stage and the results of the pre-test, the researcher will explore, and review interventions and programs of changes regard to breastfeeding behavior in Iran and other countries and search in reliable scientific databases. After that, to determine the educational content and plan educational sessions in the field of breastfeeding behavior of these mothers, a meeting will be held consisting of the research team and a group of health, gynecology, and pediatric experts from the health department of Mashhad and Ardabil University of Medical Sciences. By using the existing instructions and guidelines and the opinions of the research team and experts, the appropriate educational methods are selected to achieve the set goals according to the structures of the theory of planned behavior. To achieve each goal, the best and most applicable educational method must be determined. Based on this, at the end of this stage, the design and implementation of the plan will be determined. Then, the (educational) content will be provided to a group of mothers with gestational diabetes to ensure its comprehensibility.

### Quantitative phase methodology

Quantitative studies are regular and systematic studies in which data are collected objectively and phenomena are studied with precise and quantitative measurements. In these studies, the obtained data are tested or described with statistical techniques [23]. Interventional studies are a type of quantitative method used to test scientific hypotheses. In experimental methods (randomized controlled clinical trials and semi-experimental designs) an intervention program is used to achieve the goals of the test, which is measured through a set of predetermined indicators. In semi-experimental designs, there is a comparison group that has the same basic characteristics as the intervention group, and after the implementation of the intervention program in the intervention group, any difference between the results of the control and intervention groups is measured [27]. Semi-experimental designs facilitate the investigation of causality in situations where complete control is not possible. Pre-test-post-test design with a comparison control group is the most common design used in research [28]. In the current mixed study, the quantitative phase will aim to determine the impact of education based on the theory of planned behavior on the breastfeeding behavior of women with gestational diabetes, which includes implementation and evaluation.

### Research population

Mothers with a history of gestational diabetes and infants up to one year are covered by comprehensive health centers, and also who are eligible for the research sample.

### Inclusion criteria

Pregnant mothers over 36 weeks or with infants in the first 6 months with a history of gestational diabetes in the last pregnancy; not having a chronic or acute disease other than gestational diabetes; willing to participate in the research and answer the questions of the questionnaire with written consent; singleton pregnancy; not having a disease whose treatment interferes with breastfeeding.

### Exclusion criteria

Having breast diseases that prohibit breastfeeding; suffering from diseases whose treatment interferes with breastfeeding; mothers use of antidepressants and psychotropic drugs; mothers giving birth before the end of the fourth session of intervention; hospitalization of the baby; hospitalization of the mother; intrauterine death of the fetus.

### Sample size and sampling method

Sampling of the quantitative part of the study will be done randomly. Four health centers will be randomly selected. Two centers for the intervention group and two centers for the control group. The participants will be eligible mothers referred to the selected health centers and comprehensive health centers. After providing explanations about the study, written informed consent will be obtained. A randomized controlled method will be used regarding the purpose of the study and using the Apple system that exists in comprehensive health centers. The intervention and control groups are made up of mothers with a history of gestational diabetes covered by the mentioned centers. For blinding of the intervention, the control group will be selected from other healthcare centers and they will not receive any intervention from the research team during the study.

### Sample size

Since no similar article was found on breastfeeding in mothers with gestational diabetes, first a pilot study will be conducted to determine the sample size.

### Data analysis

After collecting quantitative data, the data will be analyzed by SPSS software 21. The normality of data distribution of quantitative variables will be evaluated using the Kolmogorov–Smirnov test. Independent t-test/Mann–Whitney will be used to compare normal/abnormal quantitative variables in two groups (inter-group) and paired t-test/Wilcoxon will be used to compare normal/abnormal quantitative variables (intra-group). A significance level of 0.05 will be considered.

**Quantitative program evaluation** In a quantitative study, the impact of the educational program for mothers with gestational diabetes will be evaluated using the “breastfeeding Drop” survey tool, which is planned based on the planned theoretical constructs. With this tool, changes in “attitude, subjective norms, perceived behavior control, intention, and behavior” will be measured and compared in two intervention and control groups.

**Tool description** The “Breastfeeding Drop” survey tool, is a valid and reliable 52-question Likert-scale tool based on the theory of planned behavior and has the domains of “attitude, subjective norm and perceived behavioral control about breastfeeding”, and was designed by Janaki in 1994. This tool is also known as the “Breastfeeding Behavior Assessment Tool”. Out of 52 items of this tool, 29 are attitudinal items, 13 items are related to subjective norms and 10 items are related to perceived behavioral control. Participants will answer each item of this questionnaire on a six-point rating scale. Based on the theory of planned behavior, attitude items as beliefs related to the consequences of a certain behavior are multiplied by the score resulting from its positive or negative evaluation [29]. The validity and reliability of this tool were done in Iran in 2015 [30].

## Discussion

Gestational diabetes is a type of carbohydrate intolerance that is first diagnosed during pregnancy [1]. Research has shown that gestational diabetes is associated with multiple prenatal and birth outcomes as well as an increased risk of adverse long-term outcomes [3]. In mothers with gestational diabetes the rate of breastfeeding is low, despite the incomparable benefits of breast milk in providing the health of mother and baby [4, 5]. This shows the necessity of investigating the breastfeeding experiences of mothers with gestational diabetes [5]. The results of studies showed that breastfeeding self-efficacy can be increased by encouraging and supporting breastfeeding by health care providers and facilitating early and continuous breastfeeding. Early breastfeeding support is important among women who are at increased risk for delayed initiation of lactation such as women with gestational diabetes [19]. The results of qualitative studies showed that women with diabetes emphasized the need for continuous counseling about breastfeeding and education as well as help to solve breastfeeding challenges [20, 21]. Considering the incomparable benefits of breastfeeding for both mother and baby, management and follow-up of breastfeeding for mothers with gestational diabetes should be one of the basic challenges for every society. In this study, the mixed approach will be used to understand mothers’ breastfeeding experience of gestational diabetes, which will be done using the directed content analysis method. Qualitative and quantitative methods can help scholars better understand mothers’ breastfeeding experiences and challenges in diabetic mothers. Breastfeeding in mothers with gestational diabetes is a multidimensional concept that is rooted in the subject of mothers and forms in the context of a society with the cultural complexities of that society. Breastfeeding is the result of a mother’s interaction with herself and her world. In this mixed study, the researcher will use data connection and integration. It means that the findings of the qualitative parts of the study will be used in the direction of revision (compilation of the educational program for mothers with gestational diabetes) based on the theory of planned behavior [25]. We hope that this research will be a small step in solving breastfeeding problems in mothers with gestational diabetes.

## Data Availability

Not applicable.
